# Contemporary (2019) prevalence of cardiovascular disease in adults with type 2 diabetes in Brazil: the cross-sectional CAPTURE study

**DOI:** 10.1186/s13098-021-00775-9

**Published:** 2022-01-10

**Authors:** Sérgio Vencio, André Gustavo Daher Vianna, Mariana Arruda Camara Ferreira da Silva, Dalton Bertolim Precoma

**Affiliations:** 1 UFG – Federal University of Goiás, Goiânia, GO Brazil; 2 ICF, Pharmaceutical Institute of Science, Av. Rio Verde, S/N - Cidade Vera Cruz, Aparecida de Goiânia, GO 74935-530 Brazil; 3 Curitiba Diabetes Center, Curitiba, PR Brazil; 4 Nossa Senhora das Graças Hospital, Curitiba, PR Brazil; 5 Novo Nordisk, São Paulo, SP Brazil; 6 Research Center at Angelina Caron Hospital, Campina Grande do Sul, PR Brazil

**Keywords:** Atherosclerosis, Brazil, Cardiovascular disease, Cross-sectional study, Prevalence, Type 2 diabetes

## Abstract

**Background:**

Type 2 diabetes (T2D) is a known risk factor for cardiovascular disease (CVD), and CVD is a major cause of mortality in patients with T2D. The CAPTURE study investigated the contemporary (2019) prevalence of established CVD in adults with T2D around the world. We report the findings from Brazil.

**Methods:**

The multinational, non-interventional, cross-sectional CAPTURE study was conducted across 13 countries from five continents. The current manuscript explores data for the CAPTURE study sample in Brazil. Standardized demographic and clinical data were collected from adults with T2D aged ≥ 18 years attending a single routine healthcare visit in primary or specialized care between December 2018 and September 2019. Data were analyzed descriptively.

**Results:**

Data from 912 adults with T2D were collected in the CAPTURE study in Brazil, with 822 patients from primary care and 90 patients from specialized care. Median (interquartile range [IQR]) patient characteristics were as follows: age 64 years (57; 71), diabetes duration 11 years (6; 19), glycated hemoglobin 7.7% (6.7; 9.1), and body mass index 29.5 kg/m^2^ (26.4; 33.5); 59% were female. The CVD prevalence and atherosclerotic CVD prevalence in the Brazil sample were 43.9% (95% confidence interval [CI] 40.9; 46.8) and 37.6% (95% CI 34.7; 40.5), respectively. The majority of patients with CVD had atherosclerotic CVD (85.8%). For the specific CVD subtypes, coronary heart disease prevalence was 27.9% (95% CI 25.2; 30.5), heart failure was 12.4% (95% CI 10.4; 14.4), cerebrovascular disease was 8.7% (95% CI 6.8; 10.5), and carotid artery disease was 3.4% (95% CI 2.3; 4.5). Glucagon-like peptide-1 receptor agonists and/or sodium-glucose co-transporter-2 inhibitors with proven cardiovascular benefit were prescribed to 15.5% of patients with CVD, compared with 18.4% of patients without CVD.

**Conclusions:**

CAPTURE was the first multinational, standardized study to provide contemporary data on CVD prevalence in adults with T2D in Brazil, and it demonstrated that almost one in two adults with T2D had established CVD. Except for carotid artery disease, the prevalence of all CVD subtypes in adults with T2D in Brazil appeared higher than the global CAPTURE prevalence.

*Trial registration* NCT03786406, NCT03811288

**Supplementary Information:**

The online version contains supplementary material available at 10.1186/s13098-021-00775-9.

## Background

The prevalence of diabetes, and its associated morbidity and mortality, has been increasing globally [1]. Although there is a paucity of contemporary population-based studies on the prevalence of diabetes in Brazil, the trend is also rising [2]. A cross-sectional study of diabetes mellitus with blood testing, conducted between 1986 and 1988, in a random sample of 21,847 individuals aged 30 to 69 years from nine large Brazilian cities, reported an overall prevalence of 7.6% [3]. More recently, the International Diabetes Federation (IDF) has estimated a national prevalence of diabetes of 11.4% among adults aged 20 to 79 years. This represents an estimated 16.8 million people currently living with diabetes, with the figure predicted to rise to 26 million by 2045. These data have resulted in Brazil’s overall ranking of fifth among the top countries with diabetes [1]. In 2014, the Brazilian Longitudinal Study of Adult Health (ELSA) in civil servants aged 35–74 years reported 19.7% of people with diabetes, out of which 50.4% were previously undiagnosed [4]. More recent data from the Risk and Protection Factors Surveillance System for Chronic Diseases by Telephone Survey (Vigitel) study in 2019 showed that the prevalence of self-reported diabetes in adults was 7.4%, indicating an increase compared with 2011, which was 5.6% [5].

Diabetes is a known risk factor for cardiovascular disease (CVD). Results of a cross-sectional study in Brazil estimating the cardiovascular (CV) risk in 1382 patients with type 2 diabetes (T2D) reported that the majority of patients were at a high risk of coronary heart disease (CHD), in particular those patients with chronic microvascular complications, or those who are overweight or obese [6]. In a separate population-based study in Brazil, a higher prevalence of metabolic syndrome was reported in patients with T2D compared with the overall population [2]. Meanwhile, results of a systematic literature review estimating the global prevalence of CVD among adults with T2D over a 10-year period (2007 to 2017) reported that CVD affected 32.2% of the 4,549,481 persons with T2D who were included in the analysis [7]. The national prevalence in Brazil was reported to be 27.5% [7]. The authors also reported that CVD accounted for half of all deaths that occurred globally during the study period [7].

Based on the increasing evidence that CVD is a major cause of mortality in patients with T2D [2, 7, 8], there has been an escalating emphasis on the management of CVD risk factors in individuals with T2D. Indeed, guidance on the prevention of CVD in patients with diabetes is available from the 2017 position statement from the Brazilian Diabetes Society (SBD), the Brazilian Cardiology Society (SBC), and the Brazilian Endocrinology and Metabolism Society (SBEM). This position statement recommends that in patients with T2D at very high risk of CVD (as defined by the presence of clinical atherosclerotic disease, with previous CV events), the addition of a glucose-lowering agent (GLA) with demonstrated CV benefit, namely a sodium-glucose co-transporter-2 inhibitor (SGLT2i) or glucagon-like peptide-1 (GLP-1) analog, can be useful to reduce CV risk [9]. CV outcome trials (CVOTs) demonstrating superiority in terms of CV safety have been reported for a number of SGLT2is and GLP-1 receptor agonists (GLP-1 RAs), compared with placebo [10–15].

The CAPTURE study (NCT03786406, NCT03811288) was a non-interventional study using a standardized methodology to assess the prevalence of CVD among adults with T2D in 13 countries across the world [16]. Here, we report the CVD prevalence data for Brazil. Additionally, the CAPTURE Brazil study sample was characterized in terms of demographics, clinical parameters, and use of GLAs and CV medication, with a focus on the use of GLAs with demonstrated CV benefit.

## Methods

### Study design

The study design of CAPTURE, a multinational, non-interventional, cross-sectional study conducted across 13 countries, has previously been described [16]. The current manuscript explores the data for the CAPTURE study sample in Brazil.

The protocol was approved by the ethics committee at each participating trial site and the study was conducted in accordance with the provisions of the Declaration of Helsinki [17], the International Society for Pharmacoepidemiology Good Pharmacoepidemiology Practices [18], and local regulations for clinical research in Brazil. All participants provided written informed consent prior to study participation.

### Site selection

Local medical affairs personnel employed by the sponsor provided information on the management of people with T2D in Brazil, including the types of physicians and practices managing patients with T2D. Information was also obtained from the national coordinating investigator and from literature searches. Sites deemed to be representative of the management of T2D in Brazil were invited to participate and to complete a feasibility questionnaire. Sites that accepted the invitation were included in the study.

### Participants

Physicians at participating sites invited consecutive adults attending a single routine healthcare visit in primary or specialized care between December 2018 and September 2019 to participate within a 90-day time period. Eligible participants were aged 18 years and older at the time of informed consent, with a diagnosis of T2D ≥ 180 days prior to providing informed consent. Exclusion criteria were a diagnosis of type 1 diabetes, congenital heart disease or malformation, previous participation in this study, mental incapacity, unwillingness, or language barriers precluding an adequate understanding or cooperation.

### Data collection

Data, including demographic, anthropometric and clinical parameters, selected medical history, GLAs, and CV medications, were collected from patients’ medical records by physicians or appropriately qualified and trained individuals. Data on medications only included those currently in use or those discontinued within ≤ 3 months. GLAs were categorized according to CV benefit, as demonstrated in CVOTs, and based on the 2020 American Diabetes Association guidelines; the GLAs with CV benefit included three GLP-1 RAs (dulaglutide, liraglutide, and semaglutide) and three SGLT2is (canagliflozin, dapagliflozin, and empagliflozin) [19].

### Objectives/endpoints of the study

The primary objective of CAPTURE was to assess CVD prevalence in adults with T2D across 13 countries and individually, including Brazil, using a standardized methodology. This report focuses on the CVD prevalence data from Brazil. Furthermore, the use of CV medications and GLAs with demonstrated CV benefit in Brazil was also assessed. CVD was defined as the presence of any of the following subtypes: cerebrovascular disease, carotid artery disease, CHD, peripheral artery disease (PAD), heart failure, cardiac arrhythmia, or aortic disease. Additionally, atherosclerotic CVD (ASCVD) was designated as a subset of CVD subtypes and included the following diagnoses: cerebrovascular disease, CHD, PAD, or carotid artery disease [20] (Additional file 1: Table S1).

### Statistical analysis

Data for the study sample characteristics (including overall CVD prevalence) for the overall CAPTURE population in Brazil, in addition to subgroups stratified according to the presence of CVD (i.e., CVD and Non-CVD), were analyzed descriptively only and not statistically due to the study design. Calculated prevalence estimates were weighted by primary care/specialist care setting within Brazil.

## Results

### Study population

Data from 912 patients with T2D were collected across 21 sites in the CAPTURE study in Brazil, with 822 patients attending primary care sites and 90 attending specialist care sites. Participating trial sites recruited an average of 43.4 patients (range: 6 to 100) (trial sites are listed in Additional file 1: Table S2).

Analysis of the data for key demographic or clinical characteristics of the overall CAPTURE Brazil sample showed that 59.0% of the population were female; median (interquartile range [IQR]) data for age were 64.0 years (57.0; 71.0), with diabetes duration 11.0 years (6.0; 19.0), glycated hemoglobin (HbA_1c_) 7.7% (6.7; 9.1), and body mass index (BMI) 29.5 kg/m^2^ (26.4; 33.5). Hypertension was present in 80.9% of the overall population and kidney dysfunction (estimated glomerular filtration rate [eGFR] < 59 mL/min/1.73 m^2^) in 35.4% (Table 1). The proportion of the overall Brazil sample with obesity was 46.4%; 10.4% of patients had macroalbuminuria, 15.7% of patients had retinopathy, 28.5% of patients had nephropathy, and 16.6% of patients had neuropathy (Table 1).

**Table 1:** Demographic and clinical characteristics of the CAPTURE study population stratified by CVD status in Brazil

Characteristic	Study population N = 912		By CVD status			
			CVD n = 400		Non-CVD n = 512	
	n	Data	n	Data	n	Data
Female	912	538 (59.0)	400	190 (47.5)	512	348 (68.0)
Age, years [IQR]	912	64.0 [57.0; 71.0]	400	67.0 [61.0; 72.0]	512	62.0 [55.0; 69.0]
Race	912		400		512	
White		610 (66.9)		275 (68.8)		335 (65.4)
Asian		19 (2.1)		12 (3.0)		7 (1.4)
Black or African American		114 (12.5)		59 (14.8)		55 (10.7)
Other		169 (18.5)		54 (13.5)		115 (22.5)
Diabetes duration, years [IQR]	912	11.0 [6.0; 19.0]	400	13.2 [7.2; 21.0]	512	10.0 [5.5; 17.0]
HbA_1c_, % [IQR]	776	7.7 [6.7; 9.1]	323	7.8 [6.8; 9.1]	453	7.6 [6.6; 9.1]
HbA_1c_, mmol/mol [IQR]	776	60.7 [49.7; 76.0]	323	61.8 [50.8; 76.0]	453	59.6 [48.6; 76.0]
HbA_1c_	776		323		453	
< 7%		250 (32.2)		91 (28.2)		159 (35.1)
7–9%		318 (41.0)		148 (45.8)		170 (37.5)
≥ 9%		208 (26.8)		84 (26.0)		124 (27.4)
FPG, mmol/L [IQR]	764	7.7 [6.2; 10.1]	310	8.0 [6.0; 10.2]	454	7.6 [6.2; 9.9]
BMI, kg/m^2^ [IQR]	907	29.5 [26.4; 33.5]	400	29.8 [26.7; 33.4]	507	29.4 [26.2; 33.7]
Obesity	907		400		507	
Without obesity		486 (53.6)		206 (51.6)		280 (55.2)
With obesity		421 (46.4)		194 (48.8)		227 (44.7)
Systolic blood pressure, mmHg [IQR]	912	130.0 [120.0; 147.0]	400	130.0 [120.0; 147.5]	512	130.0 [120; 145.5]
LDL cholesterol, mmol/L[IQR]	692	2.3 [1.8; 3.0]	283	2.1 [1.6; 2.7]	409	2.5 [1.9; 3.2]
HDL cholesterol, mmol/L [IQR]	731	1.1 [0.9; 1.4]	304	1.1 [0.9; 1.3]	427	1.2 [1.0; 1.5]
Triglyceride, mmol/L [IQR]	737	1.8 [1.2; 2.5]	305	1.8 [1.3; 2.6]	432	1.8 [1.2; 2.5]
eGFR, mL/min/1.73 m^2^	674		281		393	
> 89		166 (24.6)		46 (16.4)		120 (30.5)
> 59–89		269 (39.9)		110 (39.1)		159 (40.5)
> 29–59		193 (28.6)		99 (35.2)		94 (23.9)
≤ 29		46 (6.8)		26 (9.3)		20 (5.1)
Albuminuria	412		174		238	
Normal–mildly increased		243 (59.0)		88 (50.6)		155 (65.1)
Microalbuminuria		126 (30.6)		70 (40.2)		56 (23.5)
Macroalbuminuria		43 (10.4)		16 (9.2)		27 (11.3)
Medical history of hypertension, yes	909	735 (80.9)	399	355 (89.0)	510	380 (74.5)
Familial hypercholesterolemia, yes	742	118 (15.9)	342	59 (17.3)	400	59 (14.8)
Retinopathy	912		400		512	
Yes		143 (15.7)		85 (21.3)		58 (11.3)
Yes (referred by participant)		50 (5.5)		26 (6.5)		24 (4.7)
No		719 (78.8)		289 (72.3)		430 (84.0)
Nephropathy	912		400		512	
Yes		260 (28.5)		138 (34.5)		122 (23.8)
Yes (referred by participant)		25 (2.7)		14 (3.5)		11 (2.1)
No		627 (68.8)		248 (62.0)		379 (74.0)
Neuropathy	912		400		512	
Yes		151 (16.6)		75 (18.8)		76 (14.8)
Yes (referred by participant)		49 (5.4)		24 (6.0)		25 (4.9)
No		712 (78.1)		301 (75.3)		411 (80.3)
Smoking status	907		399		508	
Current		53 (5.8)		27 (6.8)		26 (5.1)
Previous		270 (29.8)		158 (39.6)		112 (22.0)
Never		584 (64.4)		214 (53.6)		370 (72.8)
Duration of smoking,^†^ years	321	29.0 (0.0; 63.0)	184	30.0 (0.0; 63.0)	137	20.0 (1.0; 60.0)
Physical activity,^‡^ days per week	839		361		478	
0–1		515 (61.4)		251 (69.5)		264 (55.2)
2–3		192 (22.9)		58 (16.1)		134 (28.0)
4–5		94 (11.2)		38 (10.5)		56 (11.7)
6–7		38 (4.5)		14 (3.9)		24 (5.0)

Data are n (%) or median [IQR]

BMI: body mass index; CVD: cardiovascular disease; eGFR: estimated glomerular filtration rate; FPG: fasting plasma glucose; HbA_1c_: glycated hemoglobin; HDL: high-density lipoprotein; IQR: interquartile range; LDL: low-density lipoprotein; N: number of patients in the overall Brazil sample; n: number of patients in each subgroup within the Brazil sample

^†^Only applies to participants categorized as current or previous smokers

^‡^Days with ≥ 30 min of moderate activity

### CVD prevalence

In the CAPTURE Brazil population, the weighted prevalence (95% confidence interval [CI]) of CVD was 43.9% (40.9; 46.8) (Table 2). Similarly, the weighted prevalence of ASCVD in the Brazil sample was 37.6% (34.7; 40.5).

**Table 2:** Overall prevalence estimates of CVD and its subtypes: adults with T2D in Brazil (n = 912)

CVD diagnosis	Definition of CVD diagnosis	Number of patients	Prevalence [95% CI] (%)
CVD	Cerebrovascular disease; carotid artery disease; CHD; peripheral artery disease; heart failure; cardiac arrhythmia; aortic disease	400	43.9 [40.9; 46.8]
Atherosclerotic CVD	Cerebrovascular disease; carotid artery disease; CHD; peripheral artery disease	343	37.6 [34.7; 40.5]
CHD	Myocardial infarction; stable coronary artery disease; other ischemic heart disease; past revascularization procedure	254	27.9 [25.2; 30.5]
Heart failure	Symptomatic or asymptomatic heart failure; hospitalization for heart failure	113	12.4 [10.4; 14.4]
Cerebrovascular disease	Ischemic, hemorrhagic or unspecified stroke; transient ischemic attack	79	8.7 [6.8; 10.5]
Peripheral artery disease	Asymptomatic peripheral artery disease (low-ankle branchial index [< 0.90] or pulse abolition); claudication; limb ischemia; non-traumatic amputation	76	8.3 [6.6; 10.1]
Cardiac arrhythmia and conduction abnormalities	Atrial fibrillation; atrial flutter; supraventricular or ventricular tachycardia; ventricular fibrillation. bradyarrhythmia: sinus node dysfunction or AV block	46	5.1 [3.6; 6.5]
Carotid artery disease	–	31	3.4 [2.3; 4.5]
Aortic disease	Aortic dissection or aneurysm; thromboembolic aortic disease	5	0.5 [0.1; 1.0]

Data are presented according to reducing prevalence in the CAPTURE Brazil sample

AV: atrioventricular; CHD: coronary heart disease; CI: confidence interval; CVD: cardiovascular disease; T2D: type 2 diabetes

**Fig. 1:**
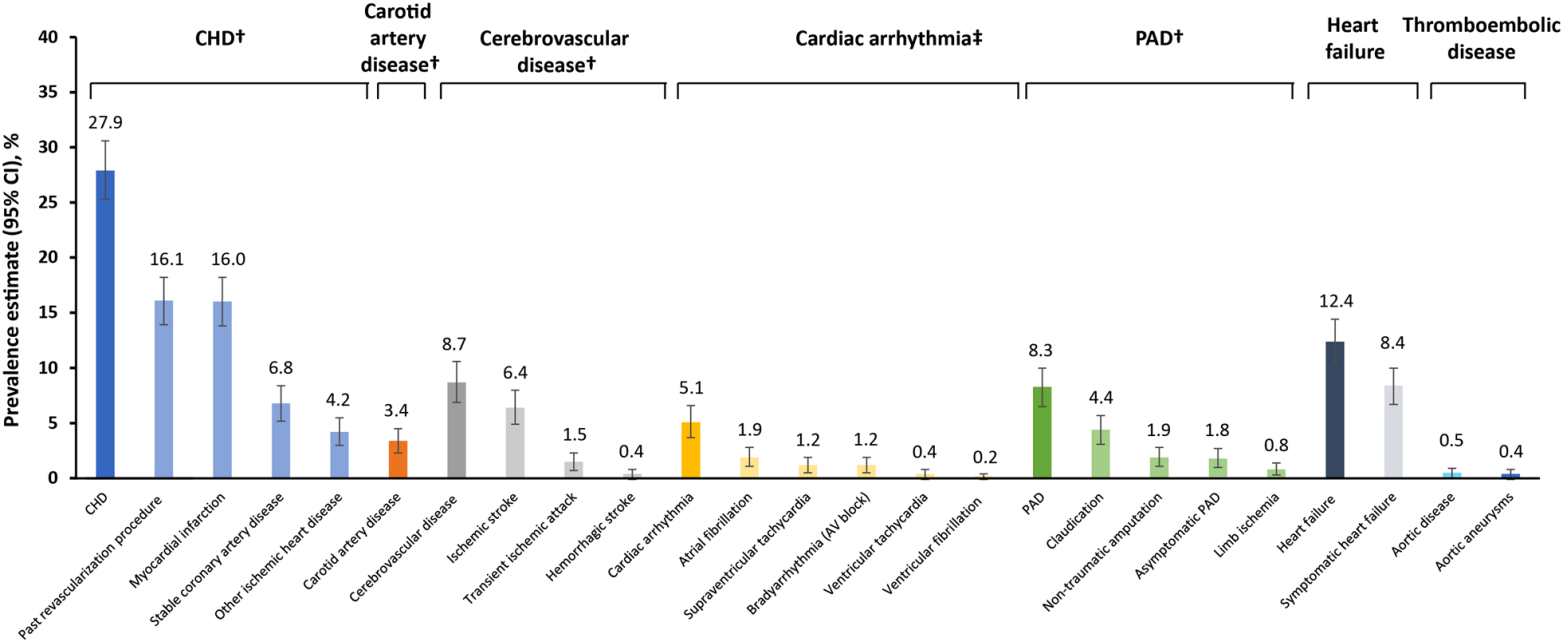
Prevalence of CVD in Brazil in patients with T2D by CVD subtype and diagnoses. Diagnoses are not mutually exclusive; one participant may have multiple diagnoses. ^†^Categorized as ASCVD. ^‡^Included conduction abnormalities. ASCVD: atherosclerotic CVD; AV: atrioventricular; CHD: coronary heart disease; CI: confidence interval; CVD: cardiovascular disease; PAD: peripheral artery disease; SND: sinus node dysfunction; T2D: type 2 diabetes

In the Brazil sample, the CVD subtypes with the highest weighted prevalence (95% CI) were CHD 27.9% (25.2; 30.5), heart failure 12.4% (10.4; 14.4), and cerebrovascular disease 8.7% (6.8; 10.5; Fig. 1). Within the cerebrovascular disease subtype, the most prevalent diagnosis was ischemic stroke (6.4%; 4.8, 7.9).

Furthermore, among the 400 patients with T2D and CVD in Brazil, 343 (85.8%) had ASCVD. Analyzing the CVD subtypes in this CVD patient group, CHD was the most common subtype (63.5% [n = 254/400], followed by heart failure (28.3% [n = 113/400], cerebrovascular disease (19.8% [n = 79/400]), and PAD (19.0% [n = 76/400]).

### Characteristics of the study population stratified by CVD status

Stratification of the CAPTURE Brazil population by CVD status revealed that the CVD subgroup, compared with the Non-CVD subgroup, was numerically older (67.0 [IQR 61; 72] vs 62.0 [IQR 55; 69] years), had a lower proportion of females (47.5% vs 68.0%), and a higher proportion with hypertension (89.0% vs 74.5%) and kidney dysfunction (eGFR < 59 mL/min/1.73 m^2^: 44.5% vs 29.0%), in addition to a higher proportion of smokers (current/previous: 46.4% vs 27.1%), although differences between the subgroups were not formally tested. Prevalence of microalbuminuria also appeared higher among the CVD versus the Non-CVD subgroup (40.2% vs 23.5%), as were the microvascular complications of retinopathy (27.8% vs 16.0%), nephropathy (38.0% vs 25.9%), and neuropathy (24.8% vs 19.7%; Table 1; not statistically analyzed).

### Use of glucose-lowering medication

In the total CAPTURE Brazil sample, GLAs were used by 98.7% of patients overall, with use being similar among the CVD and Non-CVD subgroups (98.3% and 99.0%, respectively). Oral antidiabetic drugs were used by 88.9% of the overall population, with a numerically lower proportion of patients in the CVD group receiving oral antidiabetic drugs (86.0%) compared with those in the Non-CVD group (91.2%). Insulin was used by 40.8% of the overall population, 47.0% of the CVD population, and 35.9% of the Non-CVD population (Additional file 1: Table S3). GLAs with proven CV risk reduction—GLP-1 RAs and/or SGLT2is—were prescribed to 17.1% of the overall population (15.5% in the CVD subgroup; 18.4% in the Non-CVD subgroup). SGLT2is were prescribed to 16.0% of the overall population (14.3% with CVD; 17.4% without CVD), while GLP-1 RAs were prescribed to 2.0% of the overall population (2.8% with CVD; 1.4% without CVD; Additional file 1: Fig. S1). Differences between the CVD and Non-CVD subgroups were not formally tested. A summary of the GLP-1 RAs and SGLT2is prescribed to patients in the Brazil sample is provided in Additional file 1: Table S4. Overall, 0.8%, 1.0% and 0.2% of patients were receiving dulaglutide, liraglutide and semaglutide, respectively, and 11.1% and 5.0% of patients were receiving dapagliflozin and empagliflozin, respectively (Additional file 1: Table S4).

### Use of CV medications

CV medications were used by 89.9% of the overall Brazil sample, and in 97.8% and 83.8% of the CVD and Non-CVD subgroups, respectively. The most frequently prescribed CV medications in the CVD group were statins (78.5%), acetylsalicylic acid (54.3%), and angiotensin II receptor blockers (54.0%), while the most frequently prescribed CV medications in the Non-CVD group were statins (56.4%), angiotensin II receptor blockers (44.5%), and thiazides (25.2%; not statistically analyzed, Additional file 1: Table S5).

## Discussion

CAPTURE represents the first multinational, standardized study to estimate CVD prevalence in adults with T2D. Among the 13 participating countries, Brazil had the highest recruitment, with 9.3% (n = 912/9823) of all patients recruited to the CAPTURE study, and the second highest weighted prevalence of CVD (after Israel, which had a prevalence of 50.7%). Analysis of data for the CAPTURE Brazil sample demonstrated that almost one in two adults (43.9%) with T2D had established CVD, which is a higher prevalence than that seen in the overall multinational sample (34.8%) (16).

In Brazil, ASCVD was highly prevalent among patients with T2D (37.6%; n = 343/912) and among those with T2D and CVD (85.8%; n = 343/400). Within the subtypes of CVD, CHD was the most prevalent one, with CHD, heart failure, cerebrovascular disease, and PAD being more prevalent compared with other subtypes (carotid artery disease, cardiac arrhythmia and thromboembolic disease), albeit at lower rates than ASCVD, as ASCVD is a combination of several CVD subtypes. The prevalence of all CVD subtypes were higher in the Brazil sample compared with the multinational sample, with the exception of carotid artery disease, which was lower in the Brazil sample than in the multinational sample (3.4% vs 8.4%) [16]. It is possible that patients recruited to CAPTURE in Brazil had higher numbers of complications compared with the multinational sample, as the Brazilian centers carrying out research tended to serve complex patients.

Analysis of the sample from Brazil according to CVD status showed that patients in the CVD subgroup compared with the Non-CVD subgroup were older and fewer were female. A higher proportion of the CVD group than the Non-CVD group was also observed to have hypertension, kidney dysfunction, microalbuminuria, and microvascular complications. However, the differences between the CVD subgroups were not formally tested and should, therefore, be interpreted with caution. Hypertension—a common risk factor for CVD [21]—appeared to be more prevalent in the Brazil sample than in the multinational sample (80.9% vs 70.1%, respectively), despite the higher use of blood pressure medications in the Brazil sample (use of medications for hypertension and other CVDs: 79.5% vs 61.4%, respectively [16]). Additionally, kidney dysfunction (eGFR < 59 mL/min/1.73 m^2^) was seen in more patients in the Brazil sample (35.4%) compared with the multinational sample (21.0%). The median HbA_1c_ levels in Brazil appeared slightly higher versus the multinational sample (7.7% vs 7.3%, respectively [16]), which would be consistent with the results of a previous study reporting poor glycemic control among Brazilian patients with T2D [22].

It is interesting to note the relatively low use of statins (78.5%) (and aspirin, 54.3%) in the CVD group, given the increased emphasis on lipid-lowering (and ongoing prevention of atherosclerotic events) in the 2017 guidelines [9]. Nevertheless, the median LDL levels in the CVD group are within the intermediate risk target (< 2.6 mmol/L for LDL) stipulated by the guidelines [9] and, compared with the multinational sample (64.2% for statins and 39.0% for aspirin) [16], their use is considerably higher.

Use of any GLA with proven CV benefit (GLP-1 RA and/or SGLT2i) was lower in the Brazil sample than in the overall multinational study population (18.4% vs 21.9%, respectively [16]). Moreover, the results from the Brazil sample showed that numerically fewer patients in the CVD subgroup, compared with the Non-CVD subgroup, were prescribed any GLAs with proven CV protection or an SGLT2i specifically, while slightly more in the CVD subgroup were prescribed GLP-1 RAs. Potential reasons for the relatively low rate of prescribing GLP-1 RAs, despite their proven CV benefit, might be due to their injectable route of administration, the high cost of these medications and therapeutic inertia [23, 24]. The low rate of prescription of GLAs with proven CV benefit may suggest that patients with T2D in Brazil may be managed sub-optimally in this regard. Indeed, in Brazil, a high proportion of patients with diabetes are treated solely in primary care [22], where physicians may not be well-equipped to manage many of these complex patients with established CVD; however, a larger percentage of the CVD group was prescribed antihypertensives compared with the Non-CVD group, indicating that physicians were aware of their increased CV risk and medication need.

There may also have been medication-specific reasons why the rate of the prescription of SGLT2is was lower in the Brazil sample compared with the global sample. For example, in the CAPTURE global sample, a total of 91 patients were treated with the SGLT2i canagliflozin, which has proven CV benefit [25]; however, no patient was treated with canagliflozin in the CAPTURE Brazil sample, possibly due to its label warning (contained within local package inserts/summary of product characteristics/labels) [25–27]. Furthermore, while the 2017 Brazilian guidelines recommend the use of GLP-1 RAs and SGLT-2is to reduce CV risk, some additional key information to support this recommendation was only just presented during/just after the time of data collection [10, 14].

Linked to differences in prescribing patterns, a previous sub-analysis of an online survey explored the perceptions and routines of patients with T2D and physicians in Brazil, compared with other countries (USA, UK, Spain, India, and Japan) [28]. Preventing CV events was highlighted as the primary concern of healthcare professionals, while reducing microvascular complications that impacted quality of life was the priority of patients with T2D in Brazil [28]. This discrepancy in priorities between healthcare professionals and patients with T2D in Brazil may lead to poor understanding of the condition and, consequently, a lower use of GLAs with proven CV benefit in Brazil than in the multinational study sample. Given the relatively low rates of prescription of GLAs with proven CV benefit in the Brazil sample, education should be delivered to all healthcare professionals in Brazil regarding the CV benefit of SGLT2is and GLP-1 RAs to assist in the prevention of CV events.

The CAPTURE study in Brazil had a number of limitations. There was the potential for selection bias in this cross-sectional study that may have resulted in study sites and a population sample that were not representative of the management of T2D in Brazil, and the sample size of patients with T2D in secondary care setting is small. Relative to the size of the T2D population in Brazil, the CAPTURE study sample was small (n = 912 patients). Data were not formally tested statistically, limiting the robustness of any comparisons. Finally, this non-interventional study did not screen or adjudicate for the presence of CV complications; thus, there may have been under-or over-diagnosis of CVD.

## Conclusions

The CAPTURE study provides valuable contemporary cross-sectional data on CVD prevalence in Brazilian patients with T2D, demonstrating that almost one in two have established CVD, of which the majority have ASCVD. All CVD subtypes had higher prevalence in the Brazil sample compared with the multinational sample, with the exception of carotid artery disease. Standard therapeutic strategies to ameliorate CV risk factors appear to be utilized in this population (i.e. statin and aspirin treatment), although these rates could be improved. Likewise, improvements in HbA_1c_ levels should be strived for. The results also show a lower use of GLAs with demonstrated CV benefits in the Brazil sample versus the multinational sample, despite national guidelines advocating their use in patients with T2D and ASCVD or at very high risk of CVD [9]. Providing more education to healthcare professionals to increase the discussion about the treatment of patients with T2D and CVD could help to reduce clinical inertia and improve patient outcomes.

## Supplementary Information

**Additional file 1: Table S1.** Definition of CVD diagnoses in the CAPTURE study. **Table S2.** List of participating trial sites. **Table S3.** Use of glucose-lowering agents in the CAPTURE study population stratified by CVD status in Brazil. **Table S4. **Proportion of patients receiving GLP-1 RAs and SGLT2is with proven CV benefit (Brazil). **Table S5.** Use of CV medications in the CAPTURE study population stratified by CVD status in Brazil. **Figure S1.** GLAs with proven CV benefit use in the CAPTURE Brazil sample stratified by CVD status.

## Data Availability

The datasets supporting the conclusions of this article are included within the article and its additional file. The patient level analysis data sets for the research presented in the article are available from the corresponding author on reasonable request.

## Abbreviations

ASCVD: Atherosclerotic cardiovascular disease
BMI: Body mass index
CHD: Coronary heart disease
CI: Confidence interval
CV: Cardiovascular
CVD: Cardiovascular disease
CVOTs: Cardiovascular outcome trials
eGFR: Estimated glomerular filtration rate
GLA: Glucose-lowering agent
GLP-1 RAs: Glucagon-like peptide-1 receptor agonists
GLP-1: Glucagon-like peptide-1
HbA_1c_: Glycated hemoglobin
IDF: International Diabetes Federation
PAD: Peripheral artery disease
SBC: Brazilian Cardiology Society
SBD: Brazilian Diabetes Society
SBEM: Brazilian Endocrinology and Metabolism Society
SGLT2i: Sodium-glucose co-transporter-2 inhibitor
T2D: Type 2 diabetes
