# Supplementary material for: Contemporary (2019) prevalence of cardiovascular disease in adults with type 2 diabetes in Brazil: the cross-sectional CAPTURE study
Source: Diabetol Metab Syndr. 2022 Jan 10;14:5. doi: 10.1186/s13098-021-00775-9 (PMC8751351; doi:10.1186/s13098-021-00775-9)
Supplement: Supplementary file 1 — Additional file 1: Table S1. Definition of CVD diagnoses in the CAPTURE study. Table S2. List of participating trial sites. Table S3. Use of glucose-lowering agents in the CAPTURE study population stratified by CVD status in Brazil. Table S4. Proportion of patients receiving GLP-1 RAs and SGLT2is with proven CV benefit (Brazil). Table S5. Use of CV medications in the CAPTURE study population stratified by CVD status in Brazil. Figure S1. GLAs with proven CV benefit use in the CAPTURE Brazil sample stratified by CVD status. [file 13098_2021_775_MOESM1_ESM.pdf]

Contemporary (2019) prevalence of cardiovascular disease in adults with type 2 diabetes in Brazil: the cross-sectional CAPTURE study

Sérgio Vencio, André Gustavo Daher Vianna, Mariana Arruda Camara Ferreira da Silva, Dalton Bertolim Precoma

Supplementary material/Additional file

Contents:

Table S1. Definition of CVD diagnoses in the CAPTURE study.

Table S2. List of participating trial sites.

Table S3. Use of glucose-lowering agents in the CAPTURE study population stratified by CVD status in Brazil.

Table S4. Proportion of patients receiving GLP-1 RAs and SGLT2is with proven CV benefit (Brazil).

Table S5. Use of CV medications in the CAPTURE study population stratified by CVD status in Brazil.

Figure S1. GLAs with proven CV benefit use in the CAPTURE Brazil sample stratified by CVD status.

Table S1. Definition of CVD diagnoses in the CAPTURE study

CVD subtype	Accepted diagnoses	Further optional details
Cerebrovascular disease	Ischemic stroke	–
	Hemorrhagic stroke	–
	Unspecified stroke	–
	Transient ischemic attack	–
CHD	Myocardial infarction	–
	Stable coronary artery disease	Also referred to as angina pectoris
	Other ischemic heart disease	–
	Past revascularization procedure	–
Heart failure	Symptomatic heart failure	NYHA group II–IV; LVEF (%) <40. 40–<50. or ≥50%
	Asymptomatic heart failure	NYHA group I with LVEF (%) <40. 40–<50. ≥50%. or unknown
	Hospitalization for heart failure	–
Cardiac arrhythmia and conduction abnormalities	Atrial fibrillation	–
	Atrial flutter	–
	Ventricular tachycardia	–
	Supraventricular tachycardia	–
	Ventricular fibrillation	–
	Bradyarrhythmia	Sinus node dysfunction or atrioventricular block
Aortic disease	Aortic dissection	–
	Aortic aneurysm	–
	Thromboembolic aortic disease	–
PAD	Asymptomatic PAD	Low ankle-branchial index (<0.90) or pulse abolition
	Claudication	–
	Limb ischemia	–
	Non-traumatic amputation	–
Carotid artery disease	Carotid artery disease	–

From original Table S1 from Mosenzon *et al. Cardiovasc Diabetol* 2021; doi: 10.1186/s12933-021-01344-0, under the Creative Commons Attribution License 4.0; Copyright © 2021, The Author(s).
CHD, coronary heart disease; CVD, cardiovascular disease; LVEF, left ventricular ejection fraction; NYHA, New York Heart Association functional classification; PAD, peripheral artery disease.

Table S2. Participating trial sites

Centro De Pesquisas Em Diabetes E Doencas Endocrino Metabolicas Ltda.
Sociedade Hospitalar Angelina Caron.
Loema Medicina e Bem Estar - Centro Especializado em Cardiologia Ltda.
Hospital de Clinicas da Universidade Federal do Triangulo Mineiro.
Santa Casa De Misericordia de Belo Horizonte.
Clinica e Pesquisa em Endocrinologia de Maringa SS.
Santa Casa de Misericordia de Votuporanga.
CPClin- Centro de Pesquisas Clinicas Ltda./Clinica Dr. Freddy Goldberg Eliaschewitz.
Nucleo de Pesquisa Clinica do Hospital Vera Cruz.
Hospital das Clinicas da Universidade Federal de Minas Gerais (HCUFMG).
Instituto de Estudos e Pesquisas Clinicas do Ceara (IEP).
Instituto Pro-Renal Brasil.
Hospital Sao Vicente de Paulo - Bioserv SMO.
Irmandade Sta Casa Misericordia Poa - Centro Medico.
Instituto de Molestias Cardiovasculares Tatui.
CEPIC - Centro Paulista de Investigacao Clinica e Servicos Medicos Ltda.
Clinilive (Clinica do idoso e pesquisa clínica).
Hospital e Maternidade Dr. Christovao da Gama.
Centro de Diabetes Curitiba.
Nucleo de Pesquisa Clinica do RS.
Instituto de Ciencias Farmaceuticas – ICF.

Table S3. Use of glucose-lowering agents in the CAPTURE study population stratified by CVD status in Brazil

	Study population N=912	By CVD status	
		CVD n=400	Non-CVD n=512
Any glucose-lowering agent			
Yes	900 (98.7)	393 (98.3)	507 (99.0)
No	12 (1.3)	7 (1.8)	5 (1.0)
Oral antidiabetic drug			
Any	811 (88.9)	344 (86.0)	467 (91.2)
Biguanide	740 (81.1)	306 (76.5)	434 (84.8)
Sulfonylurea	273 (29.9)	127 (31.8)	146 (28.5)
DPP-4i	180 (19.7)	63 (15.8)	117 (22.9)
SGLT2i	147 (16.1)	58 (14.5)	89 (17.4)
Thiazolidinedione	32 (3.5)	11 (2.8)	21 (4.1)
Alpha glucose inhibitor	1 (0.1)	0 (0)	1 (0.2)
Glinide	0 (0)	0 (0)	0 (0)
Insulin			
Any	372 (40.8)	188 (47.0)	184 (35.9)
Basal	365 (40.0)	185 (46.3)	180 (35.2)
Bolus	178 (19.5)	91 (22.8)	87 (17.0)
GLP-1 RA	18 (2.0)	11 (2.8)	7 (1.4)
Monotherapy			
Any	316 (34.6)	134 (33.5)	182 (35.5)
Biguanide	203 (22.3)	72 (18.0)	131 (25.6)
Insulin (any)	87 (9.5)	49 (12.3)	38 (7.4)
DPP-4i	12 (1.3)	6 (1.5)	6 (1.2)
Sulfonylurea	11 (1.2)	6 (1.5)	5 (1.0)
SGLT2i	2 (0.2)	1 (0.3)	1 (0.2)
GLP-1 RA	1 (0.1)	0 (0)	1 (0.2)
Dual therapy*			
Any	361 (39.6)	166 (41.5)	195 (38.1)
Biguanide and insulin (any)	134 (14.7)	64 (16.0)	70 (13.7)
Biguanide and sulfonylurea	114 (12.5)	57 (14.3)	57 (11.1)
Biguanide and SGLT2i	35 (3.8)	12 (3.0)	23 (4.5)
Biguanide and DPP-4i	33 (3.6)	8 (2.0)	25 (4.9)
SGLT2i and insulin (any)	6 (0.7)	3 (0.8)	3 (0.6)
Biguanide and GLP-1 RA	4 (0.4)	2 (0.5)	2 (0.4)
SGLT2i and DPP-4i	3 (0.3)	1 (0.3)	2 (0.4)
SGLT2i and sulfonylureas	3 (0.3)	2 (0.5)	1 (0.2)
Biguanides and thiazolidinediones	2 (0.2)	0 (0)	2 (0.4)
GLP-1 RA and sulfonylureas	1 (0.1)	1 (0.3)	0 (0)
GLP-1 RA and insulin, any	1 (0.1)	0 (0)	1 (0.2)
Triple therapy*			
Any	175 (19.2)	79 (19.8)	96 (18.8)
Biguanide and sulfonylurea and insulin (any)	46 (5.0)	24 (6.0)	22 (4.3)
Biguanides and sulfonylurea and DPP-4i	35 (3.8)	12 (3.0)	23 (4.5)
Biguanides and DPP-4i and insulin (any)	22 (2.4)	10 (2.5)	12 (2.3)
Biguanides and SGLT2i and insulin (any)	19 (2.1)	11 (2.8)	8 (1.6)
Biguanide and sulfonylurea and SGLT2i	17 (1.9)	6 (1.5)	11 (2.1)
Biguanide and DPP-4i and SGLT2i	14 (1.5)	4 (1.0)	10 (2.0)
Biguanides and GLP-1 RA and SGLT2i	4 (0.4)	3 (0.8)	1 (0.2)
Biguanide and sulfonylurea and GLP-1 RA	2 (0.2)	2 (0.5)	0 (0)
Biguanides and GLP-1RA and insulin (any)	1 (0.1)	0 (0)	1 (0.2)
Therapy with ≥4 glucose-lowering agents			
Any	48 (5.3)	14 (3.5)	34 (6.6)

Data are n (%).

*Reported in ≥1.0% of the overall CAPTURE study population, the CVD group or the Non-CVD group.

CVD, cardiovascular disease; DPP-4i, dipeptidyl peptidase-4 inhibitor; GLP-1 RA, glucagon-like peptide-1 receptor agonist; N, number of patients in the overall Brazil sample; n, number of patients in each subgroup within the Brazil sample; SGLT2i, sodium-glucose co-transporter-2 inhibitor.

Table S4. Proportion of patients receiving GLP-1 RAs and SGLT2is with proven CV benefit (Brazil)

	Study population N=912	By CVD status	
		CVD n=400	Non-CVD n=512
GLP-1 RA			
Dulaglutide	7 (0.8)	5 (1.3)	2 (0.4)
Liraglutide	9 (1.0)	5 (1.3)	4 (0.8)
Semaglutide	2 (0.2)	1 (0.3)	1 (0.2)
SGLT2i			
Dapagliflozin	101 (11.1)	36 (9.0)	65 (12.7)
Empagliflozin	46 (5.0)	22 (5.5)	24 (4.7)

Data are n (%).
Data represent the proportion of patients using the stated GLAs with proven CV protection as per the US FDA label (most recent label prior to 1 March 2020) and ADA guidelines (GLP-1 RAs: dulaglutide, liraglutide and semaglutide; SGLT2is: canagliflozin, dapagliflozin and empagliflozin).
None of the patients included in the CAPTURE Brazil sample were prescribed canagliflozin.
ADA, American Diabetes Association; CV, cardiovascular; CVD, cardiovascular disease; FDA, Food and Drug Administration; GLA, glucose-lowering agent; GLP-1 RA, glucagon-like peptide-1 receptor agonist; N, number of patients in the overall Brazil sample; n, number of patients in each subgroup within the Brazil sample; SGLT2i, sodium-glucose co-transporter-2 inhibitor.

Table S5. Use of CV medications in the CAPTURE study population stratified by CVD status in Brazil

	Study population N=912	By CVD status	
		CVD n=400	Non-CVD n=512
Any CV medication			
Yes	820 (89.9)	391 (97.8)	429 (83.8)
No	92 (10.1)	9 (2.3)	83 (16.2)
Medications for hypertension or other CVD			
Any	725 (79.5)	370 (92.5)	355 (69.3)
Angiotensin II receptor blocker	444 (48.7)	216 (54.0)	228 (44.5)
Beta blocker	234 (25.7)	155 (38.8)	79 (15.4)
Calcium channel blocker	224 (24.6)	124 (31.0)	100 (19.5)
Angiotensin-converting enzyme inhibitor*	192 (21.1)	97 (24.3)	95 (18.6)
Combined alpha and beta blocker	118 (12.9)	109 (27.3)	9 (1.8)
Other	43 (4.7)	28 (7.0)	15 (2.9)
Angiotensin receptor neprilysin inhibitor	6 (0.7)	6 (1.5)	0 (0)
Lipid-lowering medication			
Any	615 (67.4)	317 (79.3)	298 (58.2)
Statin	603 (66.1)	314 (78.5)	289 (56.4)
Fibrate	44 (4.8)	21 (5.3)	23 (4.5)
Ezetimibe	29 (3.2)	17 (4.3)	12 (2.3)
Other	3 (0.3)	2 (0.5)	1 (0.2)
Nicotinic acid or derivative	1 (0.1)	1 (0.3)	0 (0)
Platelet aggregation inhibitor			
Any	387 (42.4)	275 (68.8)	112 (21.9)
Acetylsalicylic acid	327 (35.9)	217 (54.3)	110 (21.5)
Dual antiplatelet therapy	49 (5.4)	48 (12.0)	1 (0.2)
Other ADP receptor inhibitor	30 (3.3)	28 (7.0)	2 (0.4)
Anti-thrombotic medication			
Any	36 (3.9)	35 (8.8)	1 (0.2)
Direct factor Xa inhibitor**	28 (3.1)	28 (7.0)	0 (0)
Vitamin K antagonist	6 (0.7)	5 (1.3)	1 (0.2)
Direct thrombin inhibitor**	2 (0.2)	2 (0.5)	0 (0)
Diuretic			
Any	393 (43.1)	209 (52.3)	184 (35.9)
Thiazide	220 (24.1)	91 (22.8)	129 (25.2)
Loop	120 (13.2)	93 (23.3)	27 (5.3)
Aldosterone antagonist	76 (8.3)	70 (17.5)	6 (1.2)
Thiazide-like	41 (4.5)	14 (3.5)	27 (5.3)
Other	2 (0.2)	0 (0)	2 (0.4)
Cardiac stimulant			
Any	9 (1.0)	9 (2.3)	0 (0)
Cardiac glycoside	9 (1.0)	9 (2.3)	0 (0)
Antiarrhythmic	13 (1.4)	13 (3.3)	0 (0)
Vasodilator			
Any	59 (6.5)	59 (14.8)	0 (0)
Organic nitrate	59 (6.5)	59 (14.8)	0 (0)
Other cardiac preparation	27 (3.0)	27 (6.8)	0 (0)
Peripheral vasodilator	1 (0.1)	1 (0.3)	0 (0)

Data are n (%).

ADP, adenosine diphosphate; CV, cardiovascular; CVD, cardiovascular disease; N, number of patients in the overall Brazil sample; n, number of patients in each subgroup within the Brazil sample.

*Renin-angiotensin-aldosterone-system blockers.

**Non-vitamin K oral anticoagulants.

Figure S1. GLAs with proven CV benefit use in the CAPTURE Brazil sample stratified by CVD status

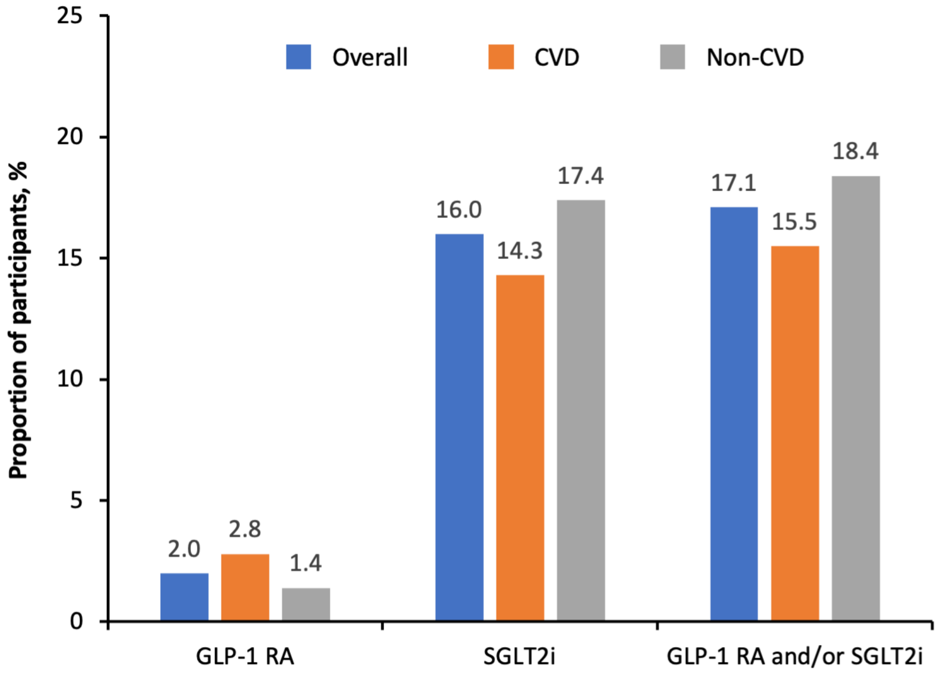

Data are proportion of participants using a GLA with proven CV protection/risk reduction per the US FDA label (most recent label prior to 1 March 2020) and ADA guidelines (GLP-1 RAs: dulaglutide, liraglutide, and semaglutide; SGLT2is: canagliflozin, dapagliflozin, and empagliflozin). None of the patients included in the CAPTURE Brazil sample were prescribed canagliflozin.
ADA, American Diabetes Association; CV, cardiovascular; CVD, cardiovascular disease; FDA, Food and Drug Administration; GLAs, glucose-lowering agents; GLP-1 RA, glucagon-like peptide-1 receptor agonist; SGLT2i, sodium-glucose co-transporter-2 inhibitor.
